# Study of the Thermal Annealing on Structural and Morphological Properties of High-Porosity A-WO_3_ Films Synthesized by HFCVD

**DOI:** 10.3390/nano9091298

**Published:** 2019-09-11

**Authors:** M. Cruz-Leal, O. Goiz, F. Chávez, G. F. Pérez-Sánchez, N. Hernández-Como, V. Santes, C. Felipe

**Affiliations:** 1 Doctorado en Nanociencias y Micro-Nanotecnologías, UPIBI, Instituto Politécnico Nacional, Ciudad de México 07340, Mexico; xaraicruz@gmail.com; 2 Departamento de Biociencias e Ingeniería, CIIEMAD, Instituto Politécnico Nacional, Ciudad de México 07340, Mexico; vsantes@ipn.mx; 3 Centro de Investigación en Fisicoquímica de Materiales, ICUAP, Benemérita Universidad Autónoma de Puebla, Edificio VAL.3, Eco Campus Valsequillo, San Pedro Zacachimalpa, Puebla 72960, Mexico; fernando.chavez@correo.buap.mx (F.C.); francisco.perezsanchez@correo.buap.mx (G.F.P.-S.); 4 Centro de Nanociencias y Micro y Nanotecnologías, Instituto Politécnico Nacional, Ciudad de México 07738, Mexico; nohernandezc@ipn.mx

**Keywords:** HFCVD, a-WO_3_, porous tungsten oxide films

## Abstract

High-porosity nanostructured amorphous tungsten OXIDE (a-WO_3_) films were synthesized by a Hot Filament Chemical Vapor Deposition technique (HFCVD) and then transformed into a crystalline WO_3_ by simple thermal annealing. The a-WO_3_ films were annealed at 100, 300, and 500 °C for 10 min in an air environment. The films were characterized by scanning electron microscopy (SEM), X-ray diffraction (XRD), micro-Raman spectroscopy, high-resolution transmission electron microscopy (HR-TEM), and UV–vis spectroscopy. Results revealed that the a-WO_3_ films were highly porous, composed of cauliflower-like structures made of nanoparticles with average sizes of 12 nm. It was shown that the effect of annealing on the morphology of the a-WO_3_ films leads to a sintering process. However, the morphology is conserved. It was found that at annealing temperatures of 100 °C, the a-WO_3_ films are of an amorphous nature, while at 300 °C, the films crystallize in the monoclinic phase of WO_3_. The calculated bandgap for the a-WO_3_ was 3.09 eV, and 2.53 eV for the film annealed at 500 °C. Finally, the results show that porous WO_3_ films preserve the morphology and maintain the porosity, even after the annealing at 500 °C.

## 1. Introduction

One of the main characteristics that nanomaterials offer is the larger specific surface area (SSA) value than that in their bulk shape. This characteristic is very important because it has opened a wide range of applications like: Adsorptive removal and photocatalytic degradation of pollutants; detection of toxic, flammable, or explosive gases; and electrochromic devices [1,2,3,4,5].

For this reason, the exploration of synthesis techniques has taken the special attention of many authors, for example: Wang et al. fabricated dispersed WO_3_ nanoparticles by the solvothermal method, and they reported the porous nanostructure with a SSA of 11.57 to 18.92 m^2^/gr [6]; Jain et al. synthesized porous tungsten oxide (PWO) by Reactive Spray Deposition Technology, and they calculated that the SSA equals 46 m^2^/gr [7]. Other methods to synthesize PWO films are sparking [8], sputtering [9], and templating [10]. Some of these techniques require sophisticated equipment or complicated processes. In this way, Hot Filament Chemical Vapor Deposition technique (HFCVD), which has been used widely to obtain diverse nanomaterials, thin and bulk films, could represent a viable technique to obtain PWO films. To the best of our knowledge, information about PWO synthesized by this technique is still limited. Nowadays, through this technique, thin semiconducting film, ceramics, and metallic materials have been produced [11], and it is considered a scalable method [12]. During an HFCVD process for metal oxides, the filament is resistively heated, provoking its oxidation due to oxygen gaseous species inside of the reactor [13]. Varying parameters, such as filament material, filament temperature, substrate temperature, filament–substrate distance (*d_f-s_*), or reactor pressure, are possible to produce uniform films with good reproducibility. Additionally, this technique offers high deposition rates, inexpensive components, and a friendly synthesis process; however, deposition of multicomponent material remains complicated [14]. Films synthesized by using the HFCVD technique have shown good ferroelectric, photoelectric, electrochromic, and catalytic properties [15,16] that have been exploited for the development of many devices, such as gas sensors [17,18], smart windows [19], diabetes monitoring [20], photocatalysis for degradation of organic dyes [21], and inkjet printing for electrochromic devices [22].

Regarding the WO_3_ synthesis films through HFCVD, a wide variety of morphologies have been obtained by controlling the surrounding gas [23], deposition time, O_2_ partial pressure [24], filament temperature [25], and substrate temperature [26]. For instance, Feng et al. deposited WO_3_ nanorods at 1400 °C under methane, hydrogen, and oxygen environments [23]; A. Jafari et al. synthesized monoclinic WO_3_ nanowalls onto silicon substrates at different temperatures (600, 700, and 800 °C) in 50 s under a mixture of argon and oxygen gases [27]; and S. Pal et al. obtained nanostructured WO_3_ films under hexacarbonyl or hexafluoride atmospheres [26].

WO_3_ is a polymorphic material that crystallizes in different phases depending on temperature. In bulk, the transformation sequence is as follows [28,29]: Monoclinic II (ε-WO_3_, < −43 °C) [30], triclinic (δ-WO_3_, −43 to 17 °C) [31], monoclinic II (γ-WO_3_, 17 to 330 °C) [32], orthorhombic (β-WO_3_, 330 to 740 °C) [33], and tetragonal (α-WO_3_, >740 °C) [34]. Nevertheless, at nanometric scale, it has been reported that both temperature and crystallite size play an important role in the phase transformation [35]. For example, when crystallite size is 60 nm, the transformation to β-WO_3_ starts at 276 °C instead of 350 °C, and transformation to α-WO_3_ starts at 676 °C instead of 897 °C [36]. A large variety of tungsten oxides, such as W_25_O_73_ (WO_2.92_), W_5_O_14_ (WO_2.8_), W_17_O_47_ (WO_2.76_), and W_18_O_49_ (WO_2.72_), exist due to the deficiency of oxygen. These oxides are also known as Magneli phases of tungsten oxide, and are characterized by the presence of crystallographic shear planes in WO_6_ octahedra [37,38,39].

This work is focused on the study of morphological and structural properties of the large surface area of PWO films synthesized by HFCVD. Characteristics of the films were studied as a function of thermal treatment from 100 to 500 °C. The results show that PWO films preserve the morphology and maintain the porosity even after the annealing at 500 °C, which makes them suitable for several applications like gas sensor devices, adsorption processes, or heterogeneous catalysis.

## 2. Materials and Methods

**Figure 1:**
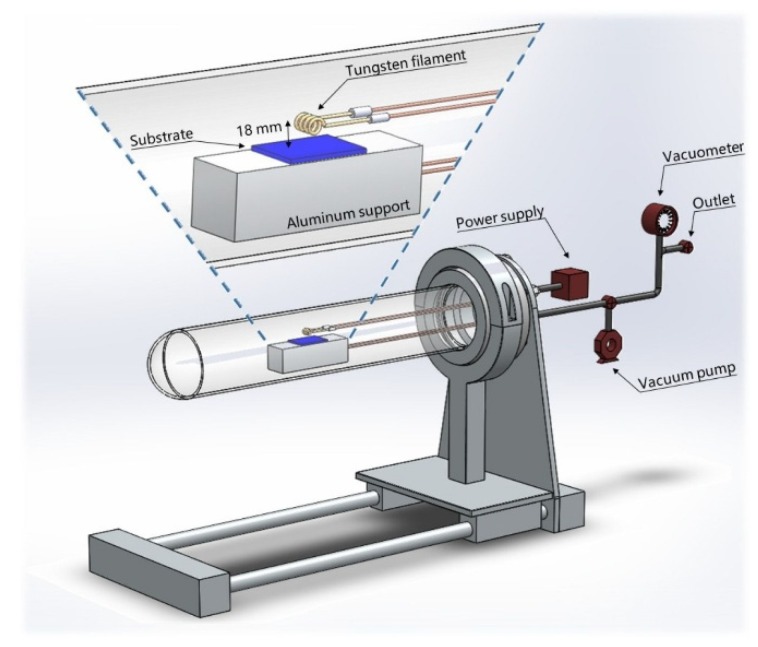
Schematic of the HFCVD reactor. Magnification shows the filament-substrate arrangement for a-WO_3_ synthesis.

A schematic of the HFCVD reactor used to synthesize the amorphous tungsten oxide (a-WO_3_) films is shown in Figure 1. It is composed mainly of a quartz tube and stainless steel parts. Tungsten filaments of 0.15 mm in diameter were used as source material. The filament temperature was estimated by using the temperature dependence of tungsten electrical resistivity, along with its electrical resistance [40]. Therefore, for a current at 2.5 A and potential at 7.5 V, 1800 °C was calculated in the filament. During the synthesis process, the pressure in the HFCVD reactor was maintained at ~10^−3^ Torr.

The *d*_f-s_ affects not only the covering area of the substrate, but morphology and composition of the film. In this context, maintaining *d*_f-s_ = 18 mm, a substrate can be covered up to 4 cm^2^ (2 × 2); however, to warrant the film homogeneity, we used silicon substrates of 1 cm^2^. The center of the substrate was placed under the center of the filament, and deposition times were fixed at 5, 10, and 15 min. Then, samples were labeled as s5m, s10m, and s15m, respectively. Before the synthesis, all the reactor components, as well as the substrates and filaments, were cleaned with a standard procedure of xylene, acetone, propanol, and deionized water, and dried with N_2_.

After synthesis, each substrate was cleaved into 4 pieces (each of 25 mm^2^), and three of them were annealed in an open room for 10 min at different temperatures: 100, 300, and 500 °C, respectively. The experimental conditions are summarized in Table 1.

The samples were analyzed using a Scanning Electron Microscope (TESCAN Vega TS513SB, with an acceleration voltage of 10 kV) to explore morphology and thickness of porous tungsten oxide films. An X-Ray Diffractometer (X’PERT PRO MRD of PANalytical) was used to obtain the structural information, carrying the measurements out with a step size [2θ] of 0.01° and scan step time of 48.19 s. A Micro-Raman Spectrometer (Horiba Jobin Yvon HR800) was used to explore the vibrational modes in the tungsten oxide films. It used a laser with a nominal power of 10.7 mW, wavelength of 633 nm, and 5 s of exposition time to avoid damaging the sample. A Transmission Electron Microscope (JEOL JEM-ARM200CF) was used to obtain the particle size distribution (PSD) and to explore the change from amorphous to the monoclinic phase of tungsten oxide. Diffuse reflectance UV–vis spectroscopy (DRUV–vis) was employed to calculate the bandgap of PWO films. Spectra were recorded from 200 to 800 using a Cary 100 UV–visible spectrophotometer. XRD, Raman and TEM equipments are located at the Centro de Nanociencias y Micro-Nanotecnologías of the IPN, México. SEM and UV-vis equipments are located at the Benemérita Universidad Autónoma de Puebla, México.

## 3. Results and Discussion

### 3.1. Morphology

**Figure 2:**
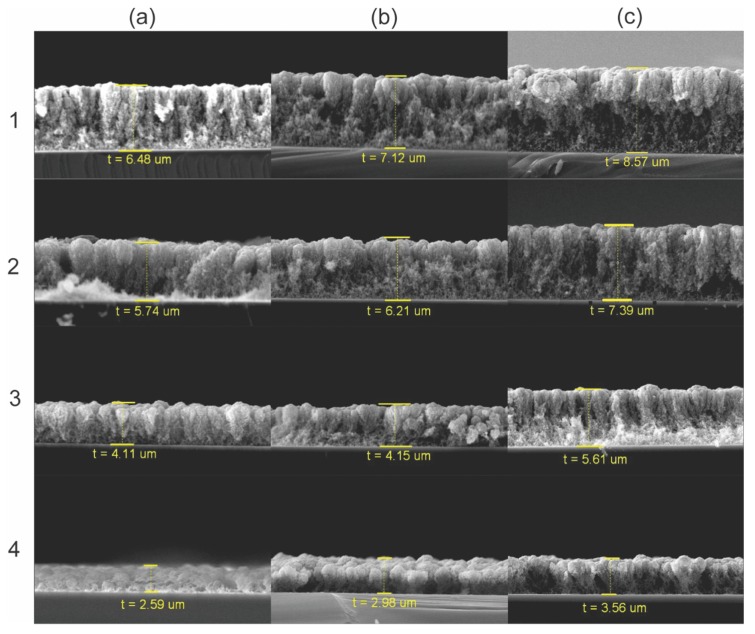
Scanning electron microscopy (SEM) images showing the thickness of the films; column (**a**): s5m, column (**b**): s10m, column (**c**): s15m; row 1: As-synthesized; annealed at: row 2: 100 °C, row 3: 300 °C, and row 4: 500 °C.

To study the effect of the annealing process on the porous films, first, the thickness of each sample was measured (column a: s5m, column b: s10m, and column c: s15m). The cross-sectional views are shown in Figure 2. From this figure, it can be observed that the thickness of the films depends on the growth time, i.e., the longer the deposition time, the thicker the film, corresponding to 6.9, 7.2, and 8.1 µm for 5, 10, and 15 min of deposition, respectively (row 1; columns a, b, c). In a previous work, it was reported that film thickness could grow up to 30 µm after 30 min of deposition, as well as that the thickness depends on the substrate temperature [41]. In Figure 2, it can be observed that the transversal morphology of the films is highly porous, composed of nanoparticles arranged in columnar structures. Additionally, it is observed that near the substrate, nanoparticles are randomly distributed, whereas near the film surface, nanoparticles are compacted, forming cauliflower-like structures. The thickness dependence on the deposition time can be attributed to a high growth ratio in the first minutes of the process, so the tungsten filament after 5 min should be almost oxidized.

Rows 2, 3, and 4 of Figure 2 show the cross-sectioned samples annealed at 100, 300, and 500 °C, respectively. In all the cases, a reduction in thickness of the films was observed as the annealing temperature increased. From Figure 2, it can be observed that when samples were annealed at 500 °C, their respective thickness was reduced by 40% in comparison with the as-synthesized one. This can be attributed to a sintering process promoted by the thermal annealing [42]. Furthermore, it is observed that samples annealed at 500 °C exhibit mainly columnar structures as opposed to randomly distributed nanoparticles. However, the surface morphology seems to be conserved; thus, sample s10m was characterized with scanning electron microscopy (SEM) to explore it.

**Figure 3:**
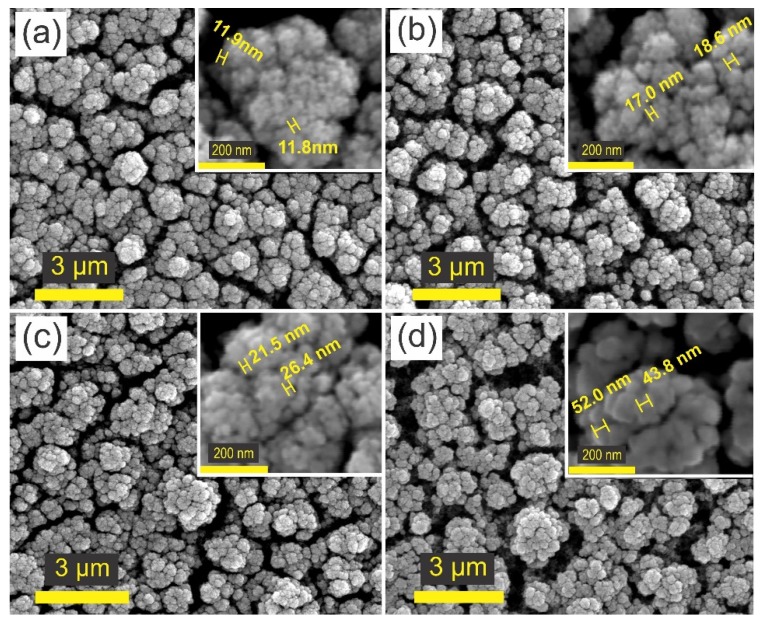
Surface morphology of the: (**a**) As-synthesized amorphous tungsten oxide (a-WO_3_) film, a-WO_3_ film annealed at (**b**) 100 °C, (**c**) 300 °C, and (**d**) 500 °C.

SEM images of Figure 3 show the surface morphology of sample s10m, before and after the annealing. It can be seen that the film exhibits a highly porous surface formed by the nanoparticle agglomerations. The nanoparticle diameter increases as the annealing temperature does, starting at 12 nm as it is shown in the inset of Figure 3a for the as-synthesized sample. For the sample annealed at 100 °C, the size of nanoparticles was around 18 nm (Figure 3b). Samples annealed at 300 and 500 °C exhibited particles of 21 and 50 nm, respectively (see insets of Figure 3c,d).

Agglomerated particles give place to cauliflower-like structures with diameters from 0.5 to 1.0 µm. These structures are separated from each other, forming cracks that were broader as annealing temperature increased.

The above results are in good agreement with those reported by Chávez et al., who, measuring through a Small-Angle X-ray Scattering technique (SAXS), observed that annealing WO_3_ powders under similar experimental conditions increased both the cauliflower-like agglomerations and the crystal size [43].

### 3.2. Crystalline Structure

**Figure 4:**
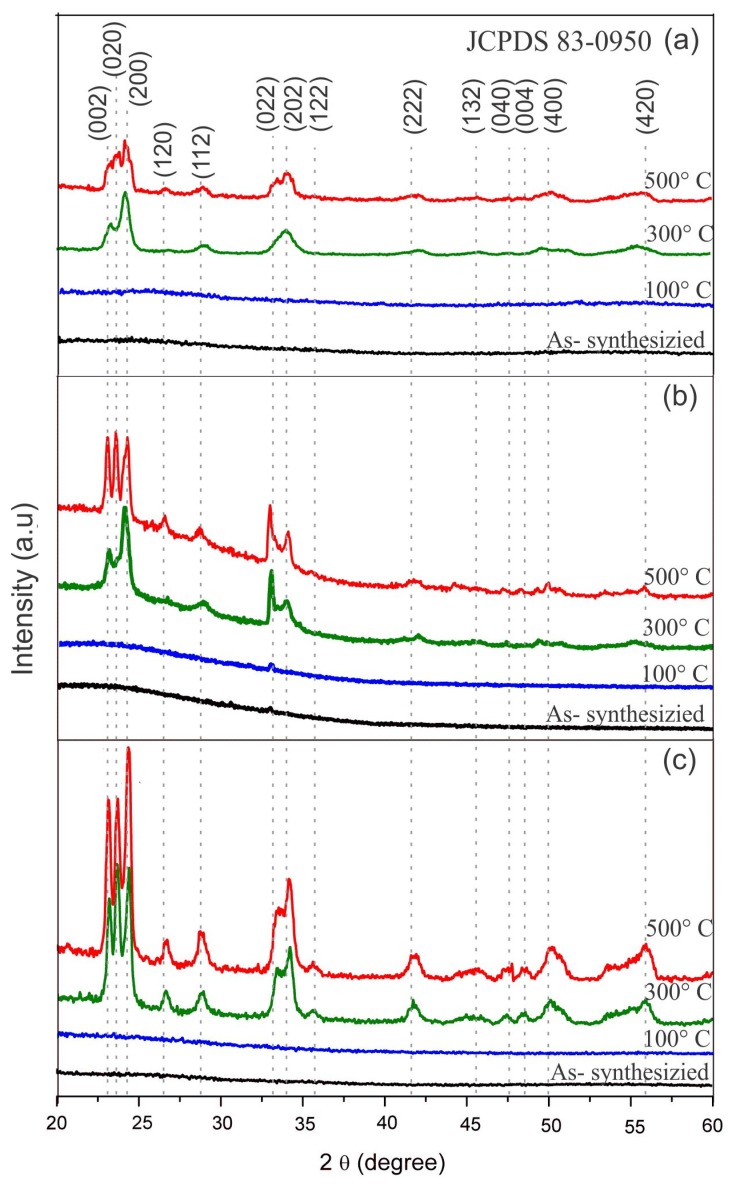
X-ray diffraction (XRD) patterns for the different annealing temperatures of the samples: (**a**) s5m, (**b**) s10m, and (**c**) s15m.

Figure 4 shows the X-ray diffraction patterns in the range of 20 to 60° for samples described in Table 1. The diffraction patterns indicate that the films maintain their amorphous phase for annealing temperatures below 300 °C, and above this temperature, the films begin to crystallize, completing their transformation to monoclinic tungsten oxide phase at 500 °C. It can also be seen that the diffraction peaks are more intense as the annealing temperature increases, being sharper in the thickest sample (Figure 4c).

Samples annealed at 300 °C and 500 °C exhibit three main peaks at 23.1, 23.7, and 24.3°, corresponding to the (002), (020), and (200) planes, respectively, of the γ-WO_3_ monoclinic phase P21/n (JCPDS 83-0950 card). As it was expected, the thermal annealing improves the crystal quality of the tungsten oxide films. Tn this case, the crystallization process is because of the reduction of structural defects from 300 °C.

Both the deposition time of the a-WO_3_ films and the annealing process play an important role in the final crystalline quality of the porous film. However, although the film thicknesses seem to be of the same order of magnitude (e.g., 2.5 to 3.5 µm for samples annealed at 500 °C), the thickest film showed better crystalline quality than the thinnest film, indicating that (1) the intensity of the peaks depends on the thickness of the films, and (2) thicker films are composed of bigger crystallites. A similar behavior has been reported elsewhere for NiO thin films [44].

### 3.3. Specific Surface Area

The average crystallite size was calculated from X-ray diffraction patterns using the Debye–Scherrer formula, given by *d = (kλ/β_hkl_cosθ)*, where *d* is the diameter of the crystallite, λ(Cu*K*α) represents the wavelength of the radiation (1.54051 Å), β is the peak width of the diffraction peak profile at half maximum, *k* is the Scherrer constant (=0.9, assuming spherical particles), and θ is the Bragg’s angle. A common way to evaluate the effective grain size as well as the degree of crystallinity of the monoclinic WO_3_ layers is by evaluating the triplet peaks in the range 20 < 2θ < 30 [45,46,47]. Notice that the annealing process conduces to an increment in the crystallite size, due to the high temperature, providing nanoparticles enough energy to coalesce into larger particles promoted by the atomic diffusion [48].

As mentioned above, the specific surface area (SSA) is a crucial parameter to evaluating the potential of the porous tungsten oxide films in the field of heterogeneous catalysis, adsorption processes, and gas sensing applications. Based on the X-ray diffraction (XRD) results, the SSA was calculated for each annealing condition by the following formula [49]:

(1) $$\mathrm{SSA}=\frac{SA_{part}}{V_{part}\times density}$$

where *SA_part_* represents surface area of the particle, *V_part_* represents the volume of the particle (considering spherical approximation for the crystallite size), and using the value of density for WO_3_ equals 7.16 g/cm^3^. The calculated crystallite sizes and SSA for samples s5m, s10m, and s15m are summarized in Table 2.

The SSA calculated for the sample s10m at 300 °C was 64.59 m^2^/gr, and at 500 °C was reduced to 29.25 m^2^/gr (Table 2). In fact, this trend was observed for each sample. It shows that when the annealing temperature increases, the SSA decreases slightly. The SSA values reported here are comparable with earlier results reported elsewhere (see Table 3).

Based on the experimental observation, we consider that the oxidation of tungsten filament is caused by the residual water, according to the following reaction [43]:

(2) $$W_{solid}+\left(3-X\right)H_{2}O_{gas}\underset{heat}{\to }WO_{3-X\left(gas\right)}+\;\left(3-X\right)H_{2\left(gas\right)}$$

The tungsten filament is heated at high temperature (~1800 °C) in an air vacuum atmosphere (~10^−3^ Torr). The tungsten oxide is formed at the filament and readily desorbed due to its high vapor pressure. The desorbed molecules are collected in the substrate surface as small nanoparticles, which imitate the cauliflower-like structures.

### 3.4. Micro-Raman Analysis

**Figure 5:**
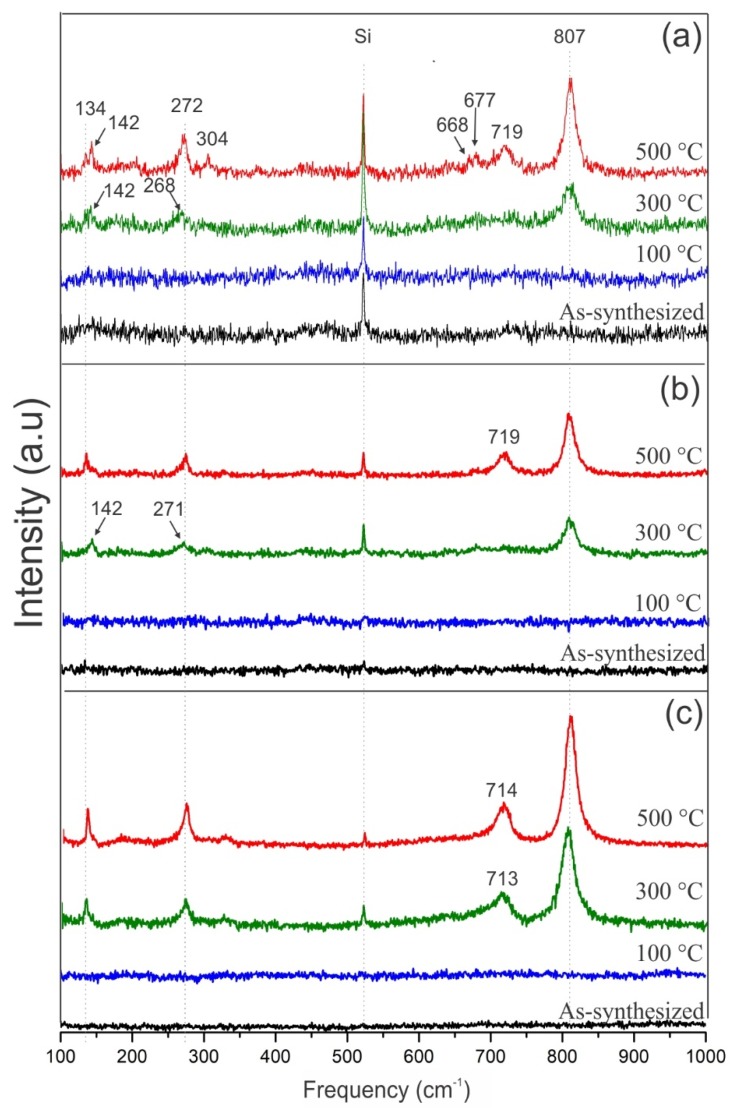
Raman spectra of the as-synthesized and the annealed samples: (**a**) s5m, (**b**) s10m, and (**c**) s15m.

Figure 5 shows the micro-Raman spectra for the as-synthesized and annealed samples in the range of 100 to 1000 cm^−1^. The signal around 521 cm^−1^ corresponds to the silicon substrate. The Raman spectra for both the as-synthesized samples and the samples annealed at 100 °C do not show any peaks, indicating that the porous films are amorphous, which is in accordance with the XRD results. These characteristic lines in Raman spectra have been reported earlier in [53], where authors observed Raman peaks only when samples were annealed above 400 °C.

For samples annealed at 300 and 500 °C, the spectra show two broad bands: A low-frequency band in the range of 100–300 cm^−1^_,_ associated with the O–W–O bending modes, and a high-frequency band in the range of 700–900 cm^−1^, associated with the W–O stretching modes. Such bands can be associated with the monoclinic phase of WO_3_ [54]_._ The peak located at 134 cm^-1^ is assigned to the lattice modes of tungsten oxide [55]. The peak around 272 cm^−1^ corresponds to bending vibration modes of the δ (O–W–O) bonds. The more intense peaks, observed around 714 cm^−1^ and 807 cm^−1^, correspond to the stretching modes of the ν (W–O) bonds [56].

From the s5m sample annealed at 500 °C, additional low intensity peaks located around 142, 304, 668, and 677 cm^−1^ rise, which can be due to the partial oxidation of tungsten, which provokes the formation of non-stoichiometric tungsten oxide (WO_3−x_) [57,58,59].

Raman spectra of samples annealed at 500 °C show slightly narrower and more intense peaks than those annealed at 300 °C, indicating that the crystallinity of the films increases gradually with the increase of the annealing temperature. The peak at 807 cm^−1^ observed for monoclinic tungsten oxide does not change as a function of annealing temperature, indicating the obtaining of highly stable crystalline porous films. A mix of γ and δ phases is discarded from this analysis mainly because it has been reported earlier that it occurs at sintering temperatures from 600 to 1000 °C [60].

The slight shift towards lower wavenumbers of peaks at 272 and 719 cm^−1^ could be due to the growth occurring in a non-equilibrium process, so the synthesized films are obtained with high stress (compressive or tensile) mainly because of the gradient temperature between filament and substrate. This shift can also be attributed to the increment in the particle size [53,61,62].

### 3.5. Transmition Electron Microscopy (TEM) Analysis

**Figure 6:**
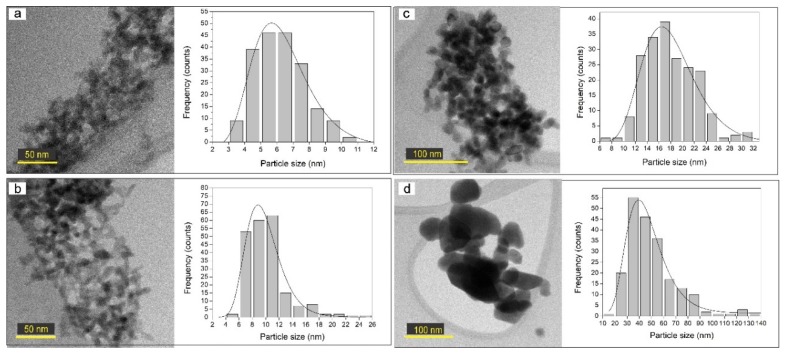
TEM images and particle size distribution of sample s10m; (**a**) as-synthesized, annealed at: (**b**) 100 °C, (**c**) 300 °C, and (**d**) 500 °C.

Each sample for TEM analysis was prepared as follows: (1) The film (over silicon substrate) was immersed in 10 mL of propanol; (2) solution was sonicated for 15 min to detach the nanoparticles from the substrate; (3) with a micropipette, a drop of the solution was taken; and (4) the drop was deposited onto a TEM grid. Figure 6 shows the TEM images, as well as the particle size distribution (PSD), for the s10m sample before and after the annealing process. The PSD was determined by using an image-processing program known as ImageJ. From the TEM images, it can be observed that the higher the annealing temperature is, the bigger the nanoparticle size, due to a sintering process promoted by the thermal annealing, which is also the reason for the film’s thickness reduction.

The graph of Figure 6a shows the PSD for the as-synthesized sample with sizes mainly from 4 to 7 nm. For the sample annealed at 100 °C, the PSD increased to 10 nm (graph of Figure 6b). Finally, the samples annealed at 300 and 500 °C presented particle sizes mostly from 12 to 22 nm and from 30 to 50 nm, respectively (graphs of Figure 6c,d). It is important to note that as the annealing temperature increases, so does the PSD, being broader (20 to 80 nm) when the sample was annealed at 500 °C.

**Figure 7:**
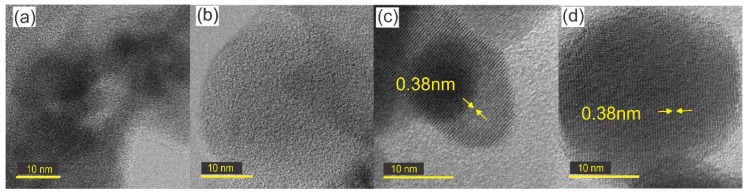
Interplanar distance of sample s10m (**a**) as-synthesized; annealed at: (**b**) 100 °C, (**c**) 300 °C, (**d**) 500 °C.

HR-TEM images (Figure 7) show that there is no evidence of crystalline domains for the sample annealed at 100 °C, nor for the as-synthesized one, which indicates the amorphous nature of the films. On the other hand, samples annealed at 300 and 500 °C exhibit crystalline nanoparticles of 15 and 30 nanometers, respectively, with interplanar distance in both cases of 0.38 nanometers along the direction (002) of the monoclinic tungsten oxide, according to the JCPDS 83-0950 card. These results are in good agreement with those previously determined with the Scherrer’s formula, summarized in Table 2.

### 3.6. UV–Vis Diffuse Reflectance and Bandgap

**Figure 8:**
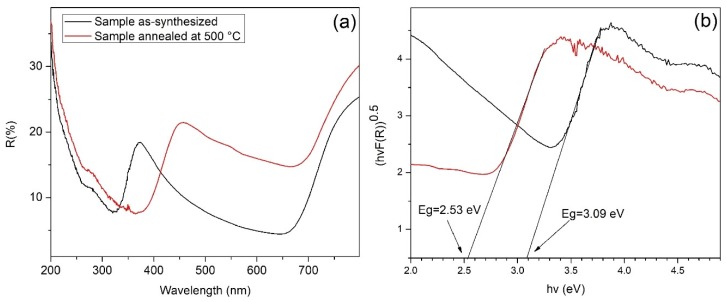
(**a**) Diffuse reflectance spectra and (**b**) Tauc plots for the as-synthesized and annealed samples.

Figure 8a shows the diffuse reflectance UV–vis spectra (DRUV–vis) recorded from 200 to 800 nm for s10m and as-synthesized samples. The black and red lines correspond to the as-synthesized samples annealed at 500 °C, respectively. The spectra revealed that the annealing increases the absorption border from 320 to 360 nm and increases the reflectance above 600 nm (see Figure 8a).

The optical bandgap (*Eg*) of both samples was extracted using the Kubelka–Munk theory, based on the following formula [63]:

(3) $$\frac{{\left(1-R\right)}^{2}}{2R}=\frac{K}{S}=F\left(R\right)$$

It is assumed in the above equation that the reflectance (*R*) and scattering coefficients (*S*) of the samples are constant for the entire wavelength range; thus, the corresponding absorption coefficients are related to the diffuse reflectance spectra, and then, it is possible to extract the band gap of the samples using the Tauc et al. method by the following equation [64]:

(4) $${\left(hvF\left(R\right)\right)}^{1/n}=A\;\left(hv-E_{g}\right)$$

Therefore, *Eg* can be determined by extrapolation of the linear region from the plot [*F(R)hv*]1/*n* versus photon energy (*hv*). For tungsten oxide, the nature of the electron transition from the valence band to the conduction band is indirect, so we use *n* = 2.

The optical bandgap values obtained for the as-synthesized and annealed samples corresponded to 3.09 and 2.53 eV, respectively (Figure 8b), which are in accordance with results reported for amorphous and crystalline tungsten oxide [65,66]. The decrease of the optical bandgap upon increasing the annealing temperature is the result of the recrystallization process in the film [67].

## 4. Conclusions

Amorphous tungsten oxide (a-WO_3_) films were deposited on silicon substrates with the HFCVD technique. The a-WO_3_ films were annealed to obtain crystalline WO_3_ films and, during this process, a film thickness reduction of around 40% was observed, which was a consequence of the sintering process. However, both the surface morphology and the bulk porosity of the films were conserved. It can be concluded that the growth time influences the film thickness in the first minutes of the process, giving place to a heterogeneous transversal morphology—as the thickness film increased (*Z*-axis), the nanoparticles were more packed in cauliflower-like structures, which was observed even after the annealing process. The heterogenous transversal morphology can explain the Raman signals observed in the thinnest film, where evidence of sub-oxides was detected. Because of the annealing treatment, the crystallite sizes were increased, and that provoked a slight decrease in the specific surface area; however, it does not affect the porosity nature of the films along the surface, nor along the *Z*-axis. In general, it is well known that HFCVD can be used to deposit metal-oxide nanostructures whenever the oxide formed on the filament has a partial pressure higher than the metal. Following that, this technique offers the possibility to probe other metals to obtain highly porous nanostructures. Moreover, the HFCVD reactor could be slightly modified by adding filaments to cover a bigger area, which would make it a scalable technique. From the presented results, it is evident that through this simple approach, it is feasible to obtain highly porous nanostructured tungsten oxide films, suitable for applications in gas sensors or photocatalysis.

## Figures and Tables

**Table 1:** Deposition time and annealing temperature for the samples.

Sample	Deposition Time (Min)	Annealing Temperature (10 Min)
s5m	5	(a) as-synthesized (b) 100 °C (c) 300 °C (d) 500 °C
s10m	10	(a) as-synthesized (b) 100 °C (c) 300 °C (d) 500 °C
s15m	15	(a) as-synthesized (b) 100 °C (c) 300 °C (d) 500 °C

**Table 2:** Average crystallite size and specific surface area (SSA) of the WO_3_ porous films for different annealing temperatures.

Sample	Temperature of Annealing	Crystallite Size	Specific Surface Area m^2^/gr
s5m	300 °C	12.88 nm	69.97
	500 °C	18.34 nm	45.78
s10m	300 °C	13.0 nm	64.59
	500 °C	28.70 nm	29.25
s15m	300 °C	23.3 nm	36.04
	500 °C	25.6 nm	32.80

**Table 3:** Comparative data for crystallite size and SSA of tungsten oxide porous films.

Synthesis Method	Temperature of Annealing	Crystallite Size	Specific Surface Area m^2^/gr	Reference
Ball-milled	600 °C	59.2 nm	3.16	[50]
Solvothermal	500 °C	47–61 nm	11.57–18.92	[6]
Coprecipitation	500 °C	66 nm	--	[51]
Sparking	500 °C	62 nm	--	[8]
Sputtering	600 °C	--	19.1	[9]
Sol-coprecipitation	600 °C	11 nm	4.7	[52]
Reactive Spray Deposition	500 °C	20–30 nm	46	[7]
HFCVD	500 °C	28.7 nm	29.25	This work
